# Supplementary material for: Hybrid Models and Biological Model Reduction with PyDSTool
Source: PLoS Comput Biol. 2012 Aug 9;8(8):e1002628. doi: 10.1371/journal.pcbi.1002628 (PMC3415397; doi:10.1371/journal.pcbi.1002628)
Supplement: Text S4 — Complete source code for the PyDSTool package (version 0.88.120504). Includes API documentation and help files linking to web pages. This file is identical to the current public release on Sourceforge.net. (ZIP) [file pcbi.1002628.s004.zip › PyDSTool/html/toc-PyDSTool.Generator.ODEsystem'-module.html]

xml version="1.0" encoding="ascii"?


ODEsystem'


# Module ODEsystem'

---

## Classes

ODEsystem  

## Variables

API  
 Continuous  
 Discrete  
 E\_COMPUTFAIL  
 E\_NONUNIQUETERM  
 HAVE\_PSYCO  
 Inf  
 LargestInt32  
 NaN  
 W\_BISECTLIMIT  
 W\_NONTERMEVENT  
 W\_NONTERMSTATEBD  
 W\_TERMEVENT  
 W\_TERMSTATEBD  
 W\_UNCERTVAL  

\_1DimplicitSolveMethods

\_all\_complex

\_all\_float

\_all\_int

\_all\_numpy\_complex

\_all\_numpy\_float

\_all\_numpy\_int

\_complex\_types

\_float\_types

\_implicitSolveMethods

\_int\_types

\_num\_equivtype

\_num\_maxmin

\_num\_name2equivtypes

\_num\_name2type

\_num\_type2name

\_num\_types

\_pytypefromtype

\_real\_types

\_seq\_types

contained  
 e  
 errmessages  
 errorfields  
 isfinite  
 notcontained  
 null\_predicate  
 pi  
 targetLangs  
 theGenSpecHelper  
 uncertain  
 warnfields  
 warnmessages  

---

[hide private]
